# Supplementary material for: MetaRibo-Seq measures translation in microbiomes
Source: Nat Commun. 2020 Jun 29;11:3268. doi: 10.1038/s41467-020-17081-z (PMC7324362; doi:10.1038/s41467-020-17081-z)
Supplement: Supplementary file 10 — Supplementary Data 7 [file 41467_2020_17081_MOESM10_ESM.zip › File2/Confidence_High_Taxonomy/66674_out.krona.html]

Javascript must be enabled to view this page.

members
magnitude
magnitudeUnassigned
count
unassigned
taxon
rank

66674\_out

3

2
3
superkingdom

3
phylum
1239

186801
class
1

186802
order
1

186806
family
1

1730
genus
1


SRS063040\_contig\_number\_26881
species
1
142586

91061
2
class

2
order
186826

1300
family
2

1301
genus
2


SRS022725\_contig\_number\_contig-100\_9680.202513
1
species
45634


SRS104647\_contig\_number\_3920
1739461
species
1
